# Correlated joint rotations in the medial foot and the definition of plantarflexion-dorsiflexion

**DOI:** 10.1186/1757-1146-5-S1-O41

**Published:** 2012-04-10

**Authors:** Thomas M Greiner

**Affiliations:** 1 Department of Health Professions, University of Wisconsin-La Crosse, La Crosse, WI 54601, USA

## Background

Every foot muscle crosses and acts upon multiple joints. The close association of foot bones and their supportive ligaments creates several closed kinematic chains. The movement of one bone necessitates the movement of several others. Terms that describe foot motion do not seem to account for these correlated motions. This presentation shows that “plantarflexion-dorsiflexion” necessitates, and therefore implies, more than just a rotation at the talocrural joint.

## Materials and methods

Observations are drawn from 10 cadaveric specimens. A rigid cluster was inserted into the tibia, talus, calcaneus, navicular, medial cuneiform and first metatarsal. An active marker camera system recorded cluster motion during manual movement of the leg through several cycles of moving the leg forward and back through the ankle joint rotation. Functional Alignment [1] processing of the data determined rotational patterns, and axis orientations, associated with the subtalar, talonavicular, medial cuneonavicular and first cuneometatarsal joints. Motion patterns about of these joints were examined as a function of talocrural plantarflexion-dorsiflexion.

## Results

**Figure 1:**
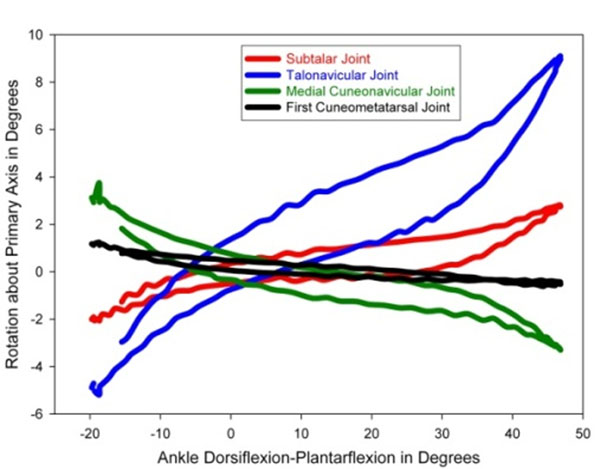
Rotational motion patterns of the medial foot joints responding to talocrural plantarflexion-dorsiflexion.

**Figure 2:**
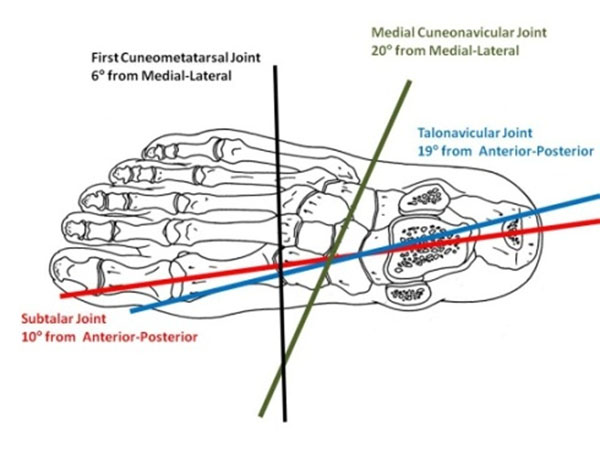
Orientation of the rotational axes when projected onto a horizontal plane.

Revealed joint rotations (Figure 1) shows how medial intrinsic foot joints respond to talocrural plantarflexion-dorsiflexion. Orientation of the joint axes (Figure 2) shows that the more proximal joints rotate about anterior-posterior orientated axis, while the axes of the distal joints return to the roughly medial-lateral axis orientation of the driving action.

## Conclusions

The joint motions presented here could be described as plantarflexion-dorsiflexion rotations, inasmuch as that term describes the results of the driving action. However, plantarflexion-dorsiflexion typically describes rotations about a medial-lateral axis [2]. Therefore, the term does not apply to all the observed rotations. In the experimental setting we can isolate joint motions and describe them with specific terms. However, in a normal functioning human no foot joint moves in isolation. Until this contradiction can be resolved, we currently have no unambiguous definition of foot plantarflexion-dorsiflexion.
